# Voltage-matrix nanopore profiling for the discrimination of protein mixtures

**DOI:** 10.1039/d5sc05182g

**Published:** 2025-09-23

**Authors:** Ryo Akita, Artem Lysenko, Keith A. Boroevich, Tatsuya Yokota, Daiki Kawai, Ryo Iizuka, Tatsuhiko Tsunoda, Sotaro Uemura

**Affiliations:** a Department of Biological Sciences, Graduate School of Science, The University of Tokyo 113-0033 Japan uemura@bs.s.u-tokyo.ac.jp; b RIKEN Center for Integrative Medical Sciences Yokohama 230-0045 Japan; c Department of Computer Science, Nagoya Institute of Technology 466-8555 Japan; d Department of Computational Biology and Medical Sciences, Graduate School of Frontier Sciences, The University of Tokyo 113-0033 Japan

## Abstract

Solid-state nanopores are attracting attention as a label-free method for detecting diverse physical properties of biomarkers. However, improving molecular discrimination in complex biological samples remains a major challenge, partly due to uncertainty in selecting optimal measurement conditions. We developed a Voltage-Matrix Analysis that visualizes classification accuracy across multiple voltages using machine learning. We measured two tumor markers (CEA and CA15-3) individually and in mixtures using solid-state nanopores, applying Random Forest and Support Vector Machine classifiers. Overfitting occurred when baseline-involving features were used, necessitating optimization of the feature set, which led to voltage-independent high classification performance. For mixed samples, we estimated actual molecular ratios by combining classification probability histograms with detection frequency correction. We further tested mouse serum with and without centrifugation and achieved notable classification accuracy. These findings suggest that voltage-dependent structural changes influence molecular discrimination, and that our method may aid diagnosis of diseases lacking known biomarkers by identifying specific molecular population shifts.

## Abstract 2

Voltage-matrix nanopore profiling combines multi-voltage nanopore recordings with machine learning, enabling robust discrimination of protein mixtures and serum samples.

## Graphical abstract

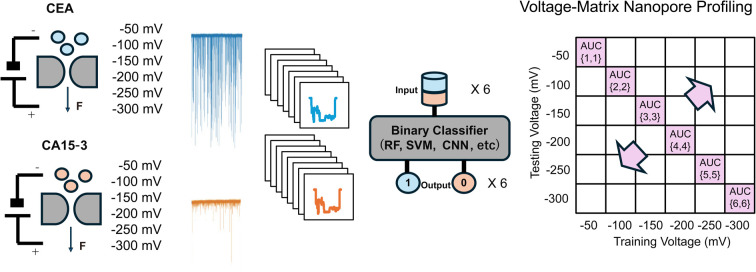

## Introduction

Accurate and high-resolution discrimination of specific molecular species and their structural or binding states from complex biological mixtures remains a major challenge in analytical chemistry, diagnostics, and drug discovery. Protein biomarkers, particularly in the context of cancer, often exist not as single molecular species but as heterogeneous states or complexes that reflect pathophysiological conditions. The ability to differentiate these molecular states in a label-free, real-time manner holds great promise for next-generation diagnostic tools.^1–4^ However, conventional technologies such as mass spectrometry, enzyme-linked immunosorbent assay (ELISA) face significant limitations in resolving such structural diversity or dynamic interactions, particularly in complex and unlabelled mixtures.^5,6^

Solid-state nanopores (SSNs) have emerged as powerful platforms for single-molecule sensing, offering high temporal resolution, minimal sample requirements, and label-free detection. The ionic current blockade signals produced during molecular translocation reflect a range of physicochemical features including size, charge, and conformational flexibility, making nanopores particularly well suited to capture subtle structural differences and dynamic binding events.^7–9^ These signals are influenced by a combination of electrophoretic, diffusive, and electroosmotic forces acting upon the molecule within the nanopore environment.^10^ While this approach has shown significant success in nucleic acid sequencing, its application to proteins and their complexes is still evolving.^11–14^

Despite these advantages, SSN-based classification faces two fundamental challenges. First, most studies rely on measurements taken under a single experimental condition (*e.g.*, fixed voltage), which limits the generalizability of classification models trained under such settings.^15–17^ Translocation signals are highly sensitive to applied voltage and buffer conditions, and models developed under one condition often fail when tested under others. Traditional approaches to SSN data analysis have generally not leveraged the ability of nanopores to capture dynamic molecular features such as structural fluctuations, conformational plasticity, or state transitions arising from complex formation. Recent studies have demonstrated that ionic current signatures can indeed report on such conformational heterogeneity,^18,19^ highlighting the latent capability of nanopores. However, these advances remain underutilized in classification frameworks, which typically rely on single-condition datasets and emphasize static size/charge readouts. Si and Aksimentiev theoretically established that ionic currents through a nanopore can report on a protein's folding state with single-molecule resolution, laying a framework for monitoring conformational transitions using SSNs.^20^ More recently, Zhou *et al.* demonstrated single-molecule protein detection directly from individual cell extracts using silicon nitride SSNs and a nanopore electrophoretic driver to enhance capture, resolving conformational changes of a photoswitchable protein in complex mixtures.^21^

Complementary to these advances, prior machine learning applications to SSN data have predominantly used feature-engineered classifiers such as support vector machines (SVMs) and Random Forest trained under a single biasing condition, leveraging dwell time, blockage amplitude, and waveform descriptors; more recently, convolutional neural network (CNN)-based models have been explored on raw current traces or spectrograms. While effective within the fixed condition, these approaches generally do not test cross-voltage generalization, leaving room for baseline-driven shortcuts.

To overcome these limitations, we propose a new strategy that treats the applied voltage not merely as a static measurement condition but as an active probe to reveal voltage-dependent translocation behaviors. Specifically, we introduce a framework called Voltage-Matrix Nanopore Profiling, in which datasets collected under multiple voltages are used to train and evaluate machine learning classifiers across all voltage combinations. This approach results in a classification performance matrix (voltage matrix) that allows visualization of classification robustness and cross-voltages generalizability.

**Scheme 1:**
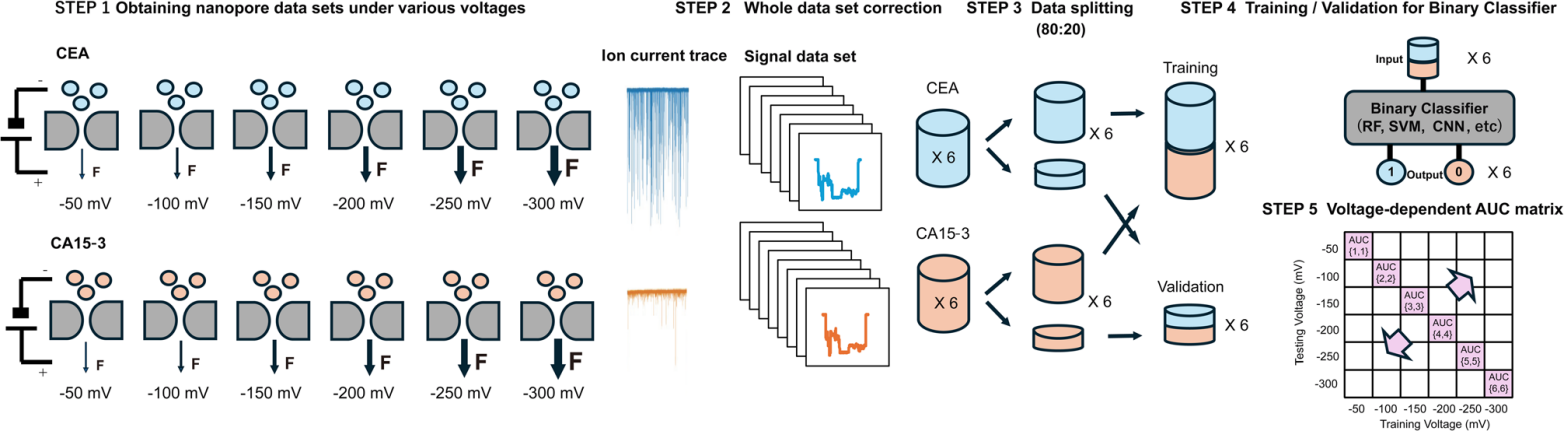
Workflow of voltage-matrix classification for solid-state nanopore sensing. Nanopore ionic current traces are acquired for two protein biomarkers (CEA and CA15-3) under six different applied voltages (−50 mV to −300 mV). In Step 1, “F” denotes an external force applied to assist molecular translocation under each voltage condition. After signal preprocessing and feature extraction (Step 1–2), the data are split into training and validation sets (Step 3). Binary classification models—including Random Forest (RF), Support Vector Machine (SVM), and convolutional neural network (CNN)—are trained at each voltage condition and validated across all voltages (Step 4). The classification performance (AUC) is summarized as a voltage-dependent matrix (Step 5), revealing the voltage-resolved robustness and generalizability of molecular discrimination.

A key aspect of our approach is to exploit the applied voltage as an active analytical axis to reveal hidden molecular properties, such as voltage-induced structural plasticity and intrinsic molecular distinctiveness. By systematically varying the voltage conditions and analysing classification performance across a matrix, we aim to capture how molecular identity, and structural states influence translocation behaviors. This voltage-matrix nanopore profiling framework holds promise for improving the resolution of complex biological mixtures, as schematically outlined in Scheme 1.

In this study, we applied this framework to three model systems: CEA, CA15-3, and the CEA–aptamer complex. We first constructed and compared their voltage-matrix classification profiles, revealing distinct patterns of robustness and voltage sensitivity. We then analyzed mixed samples to demonstrate how the approach can estimate molecular composition and highlight translocation bias. We also performed classification experiments on mouse serum samples before and after centrifugation under different pore size conditions, demonstrating the applicability of our proposed voltage-matrix method.

## Results and discussion

### Voltage-dependent nanopore profiling of CEA and CA15-3

To explore the feasibility of a voltage-matrix-based classification strategy, we first analyzed the nanopore translocation behavior of two cancer biomarkers, carcinoembryonic antigen (CEA) and cancer antigen 15-3 (CA15-3).

The molecular sizes of CEA and CA15-3 were estimated to be approximately 5–7 nm and 10–20 nm in diameter, respectively. These estimates are based on previous reports that CEA is a compact glycoprotein with a molecular weight of around 180–200 kDa,^22^ whereas CA15-3, a soluble form of the highly glycosylated MUC1 antigen, exhibits considerable size variability due to extensive glycosylation, typically extending to 10–20 nm.^23^

We reasoned that nanopores with a sufficient diameter are required to allow both molecules to translocate, and thus used SSNs with an estimated diameter of ∼12 nm fabricated *via* dielectric breakdown.

In machine-learning-based nanopore classification, the primary objective is to capture features originating from the target molecule. However, if the nanopores themselves vary in shape or size across experimental sets, classifiers may inadvertently learn pore-specific rather than molecule-specific features. To avoid this, we designed experiments in which only the target molecule was substituted while keeping all other conditions constant.

Time-resolved ionic current traces were recorded under six voltage conditions ranging from −50 mV to −300 mV (Fig. S1). A clear voltage-dependent increase in translocation event frequency was observed (Table S1). At −50 mV, the capture rates for CEA and CA15-3 were nearly identical. However, as the voltage increased, CEA showed a progressively higher frequency, with nearly a two-fold difference compared to CA15-3 at −300 mV. This result suggests that electrophoretic mobility differences between the two proteins become more pronounced with increasing electric field strength.

The isoelectric point (pI) of CEA is reported to be approximately 4.5,^22^ while CA15-3, being a heavily glycosylated mucin-type protein, lacks a well-defined pI. Nonetheless, previous reports suggest that its pI may lie within the range of pH 3 to 5.^24,25^

Given that the capture frequency of CEA exceeded that of CA15-3 under our measurement conditions (pH 8.0), the effective electrophoretic force acting on CA15-3 should be weaker than that of CEA. Furthermore, the extensive glycosylation of CA15-3 is known to affect its electrophoretic mobility, as observed in SDS-PAGE analyses,^23^ potentially further reducing its capture efficiency independent of pI. Thus, it is reasonable to assume that both the lower effective charge and the bulky glycosylated structure of CA15-3 contribute to its lower capture frequency compared to CEA.

From these traces, individual translocation signals were extracted, and two key features—dwell time and current blockage amplitude (Δ*I*)—were calculated for each event. These were visualized as two-dimensional scatter plots for each voltage condition (Fig. S2). Although the distributions of CEA and CA15-3 were largely distinguishable, overlapping regions remained, indicating the necessity of multivariate and machine-learning-based classification methods.

**Fig. 1:**
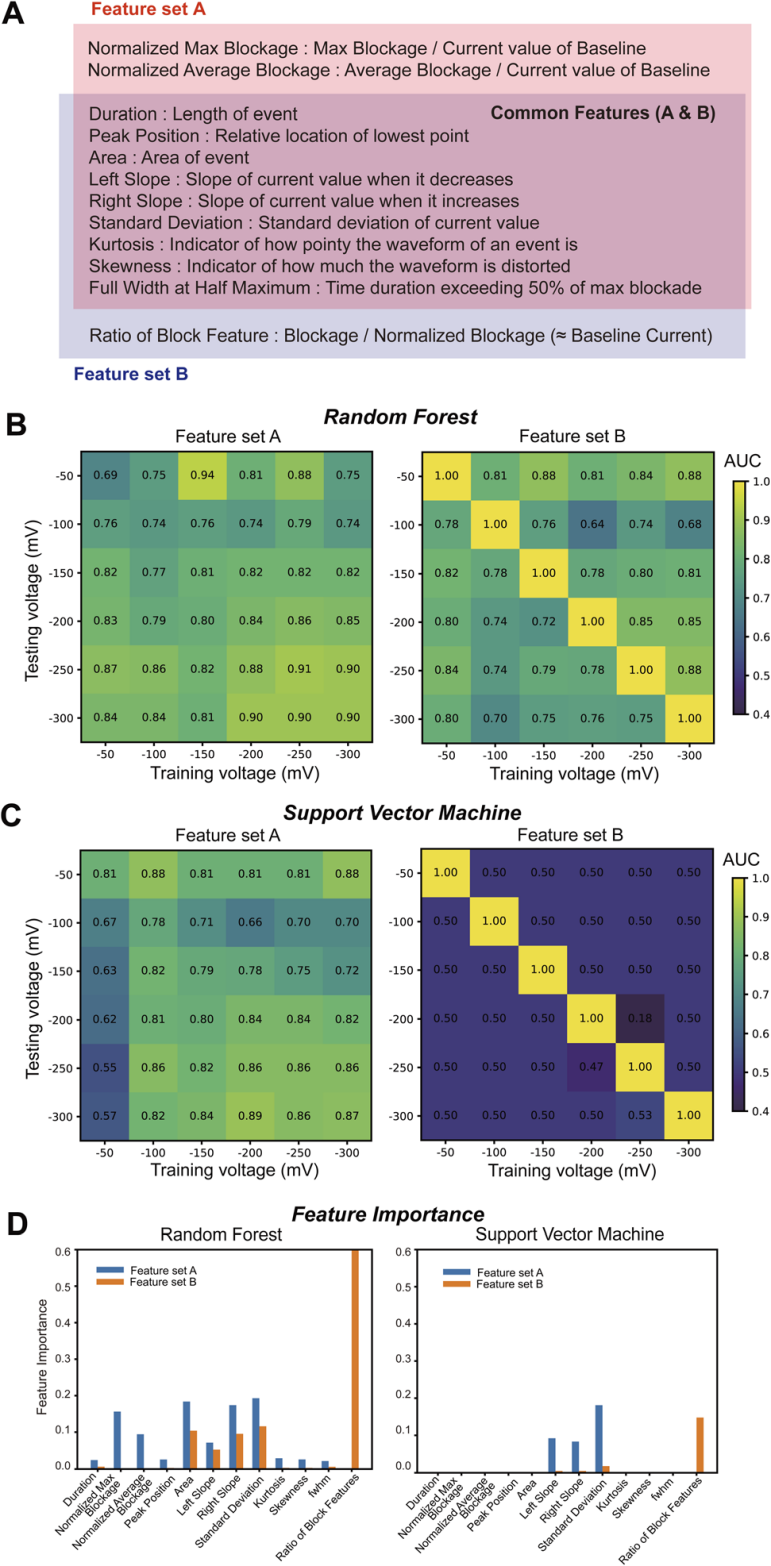
Voltage-matrix-based classification of CEA and CA15-3 using two different feature sets and machine learning algorithms. (A) The overview of the two feature sets (feature set A and feature set B) used for signal classification. The pink region shows features exclusive to feature set A, the blue region corresponds to those exclusive to feature set B, and the overlapping red-blue region indicates common features shared by both sets. (B) AUC heatmaps representing the classification performance of RF models trained and tested at different voltages, using feature set A (left) and feature set B (right). Diagonal cells (same train–test voltage) probe same-condition performance, whereas off-diagonal cells probe cross-condition generalization; diagonal-only inflation when using baseline-dependent features indicates overfitting to acquisition conditions rather than molecule. See SI Table S2 for feature taxonomy. (C) AUC heatmaps of Support Vector Machine (SVM) classification performance across the same voltage matrix conditions as in (B). Color bars represent AUC values from 0.4 (dark blue) to 1.0 (yellow), with diagonal elements corresponding to models trained and tested at the same voltage. (D) Feature importance profiles for −300 mV training data. Bar graphs show the relative importance of each feature used by the classifier during training. The left panel corresponds to the RF classifier, and the right panel to the SVM. Blue bars represent features from feature set A, and orange bars represent those from feature set B.

To assess the separability of CEA and CA15-3 signals under different voltage conditions, we first defined two sets of features: feature set A excluding baseline-dependent features, and feature set B including them (Fig. 1A). These feature sets were then used to construct voltage-matrix classification models using two supervised machine learning algorithms: Random Forest (RF) (Fig. 1B) and Support Vector Machine (SVM) (Fig. 1C). For each model, classifiers were trained on data acquired at a single voltage and tested on data from all other voltages.

The resulting of area under the curve (AUC) values were organized into 6 × 6 matrices (Fig. 1B and C), where the classification performance was evaluated using the area under the receiver operating characteristic curve (AUC). In a voltage matrix, diagonal cells represent train–test pairs under the same measurement condition (same applied voltage, pore, and buffer), and thus provide a baseline for within-condition performance. Off-diagonal cells instead evaluate cross-condition generalization, *i.e.*, how well a model trained under one voltage can classify events recorded under another. This distinction guides interpretation: robust molecular features should sustain performance both on- and off-diagonal, whereas setup-specific signatures are only valid on the diagonal.

When baseline-dependent features (*e.g.*, absolute blockade amplitude Δ*I*, open-pore current *I*_0_, baseline root mean square (RMS) noise, or slow drift/offset) are included, classifiers can exploit stable condition signatures such as the absolute current level of a given pore. This inflates diagonal AUC values because training and testing share the same baseline. However, when tested across different voltages or devices, these baseline distributions shift, leading to sharp drops in off-diagonal performance. In other words, the model is learning setup characteristics rather than molecular properties. To make this distinction transparent, we aligned feature nomenclature with Table S2 (event-intrinsic *vs.* baseline-dependent) and highlight that only baseline-agnostic feature sets maintain consistently high off-diagonal performance. For feature set A, both models showed generally high classification performance across the matrix, with particularly strong results along the diagonal (*i.e.*, matching train and test voltages) (Fig. 1B and C, left). In contrast, feature set B yielded perfect AUC scores (1.0) only along the diagonal. For a full taxonomy of the features used, including their classification and set membership, see Table S2.

This strongly suggests overfitting due to baseline features, prompting us to extract feature importance scores (Fig. 1D). As expected, feature set A exhibited a diverse distribution of importance scores, while feature set B was heavily biased toward baseline-dependent features, indicating over-reliance on these features.

These findings imply that even when sample replacement is conducted within the same experimental setup, baseline fluctuations still occur, and the inclusion of baseline-dependent features becomes a significant obstacle for molecule-specific classification.

We further performed classification using a convolutional neural network (CNN) (Fig. S3). Although high AUCs were again obtained irrespective of voltage, it remains unclear how the CNN handled baseline information, making its suitability as a classifier less straightforward to interpret. Contrary to our initial expectations that classification performance would significantly degrade when training and testing were conducted under different voltage conditions, all algorithms maintained relatively high AUC values across the matrix, despite some variation. This robustness suggests that the classifiers—regardless of model type—were able to learn signal features that are largely invariant to applied voltage.

These findings imply that, in the case of CEA and CA15-3, classification is driven more by voltage-independent structural or shape-related differences than by voltage-induced signal transformations. Such features may include size, shape, and rigidity-related properties that persist across varying electric fields.

### Voltage-dependent nanopore profiling of CEA and CEA–aptamer complex

We next examined the ionic current traces for CEA, aptamer alone, and the CEA–aptamer complex, as shown in Fig. S4. Notably, the aptamer alone—even at a high concentration of 1 μM—produced virtually no detectable signals. This absence of translocation events can be explained by the small molecular size and rapid translocation kinetics of the short single-stranded DNA aptamer relative to the ∼12 nm pore size. Under our measurement conditions, the aptamer likely passes through the nanopore too quickly and with insufficient current blockage to be detected.

**Fig. 2:**
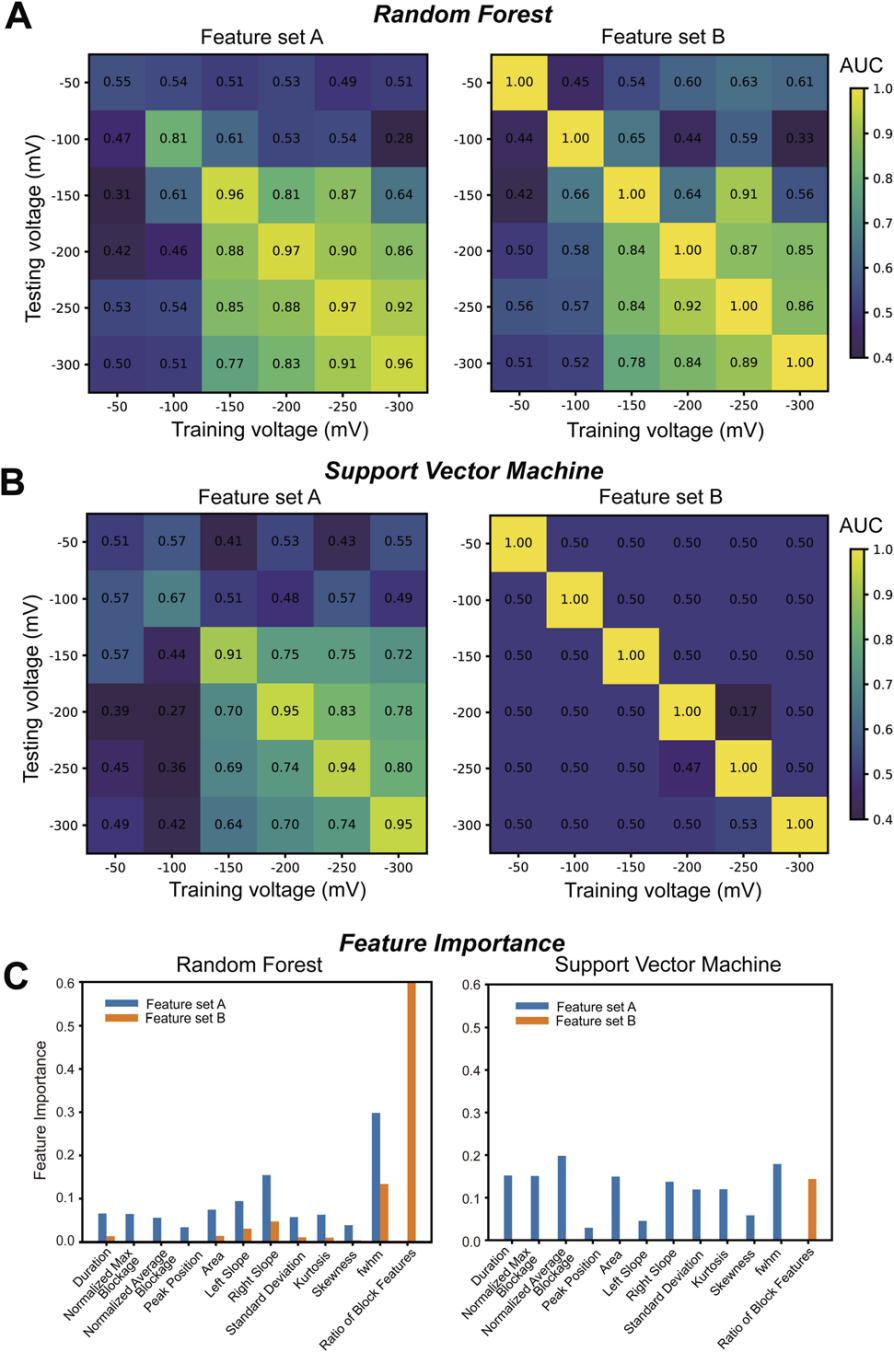
Voltage-matrix-based classification performance for CEA and CEA–aptamer complexes. (A) Heatmaps of area under the curve (AUC) values for RF classification between CEA and CEA–aptamer complex across different combinations of training and testing voltages. (Left) Results using feature set A. (Right) Results using feature set B. (B) AUC heatmaps of SVM classification under the same conditions as (A), for both feature sets A and B. Diagonal values represent classification performance when training and testing were performed at the same voltage, whereas off-diagonal elements reflect cross-voltage generalization. (C) Feature importance of individual features at −300 mV. The bar plots indicate the relative contribution of each feature to classification performance at −300 mV, as calculated by the RF (left) and SVM (right) models. Blue bars represent feature set A, and orange bars represent feature set B.

Subsequently, we compared the event capture frequencies between CEA and the CEA–aptamer complex. As summarized in Table S2, the event frequencies were generally comparable across all voltage conditions. In both RF and SVM models, feature set B exhibited signs of overfitting due to baseline-related bias, consistent with the findings in Fig. 1, Fig. 2A and B, right. In contrast, when using feature set A, high classification performance was achieved when the training and testing voltages were matched, with AUC values exceeding 0.95 (Fig. 2A and B, left).

These results strongly suggest that aptamer binding induces distinguishable structural or dynamic changes in the nanopore signal profiles. Although our measurements were conducted under high-ionic-strength conditions (3.6 M LiCl), we verified that the CEA–aptamer complex was stably formed, as supported by multiple lines of evidence. First, two-dimensional scatter plots revealed a clear shift in the signal distribution of the CEA–aptamer complex compared to CEA alone, particularly under high voltage conditions (Fig. S5). Second, atomic force microscopy (AFM) measurements (Fig. S6) demonstrated an increase in the particle size of CEA from approximately 20–30 nm to 40–50 nm after aptamer incubation, directly supporting complex formation. Although these values substantially exceed the ∼12 nm diameter of the nanopore used in this study, previous reports have shown that even globular proteins with apparent diameters of 20–40 nm as observed by AFM can translocate through nanopores of ∼10–12 nm diameter. This discrepancy is likely due to the difference between hydrated particle size on a substrate and the conformation adopted during translocation, which can involve partial unfolding or deformation under the influence of the electric field.^26^

Unlike the results for CEA *versus* CA15-3 shown in Fig. 1, the classification performance for feature set A dropped significantly when training and testing voltages differed (Fig. 2A and B, left). This suggests that the CEA–aptamer complex undergoes voltage-dependent conformational or interaction changes, which compromise the generalizability of classification. These findings indicate that while aptamer binding occurred, it introduced structural variability that is sensitive to the applied voltage—variability that can be captured through voltage-matrix nanopore profiling.

### Mixed sample classification

We next analyzed a 1 : 1 mixture of CEA and CA15-3. Representative ionic current traces are shown in Fig. S3. The capture frequency for the mixed sample approximated the sum of the individual frequencies of CEA and CA15-3 under each voltage condition (Table S1), suggesting minimal interaction between the two molecules and successful co-detection.

**Fig. 3:**
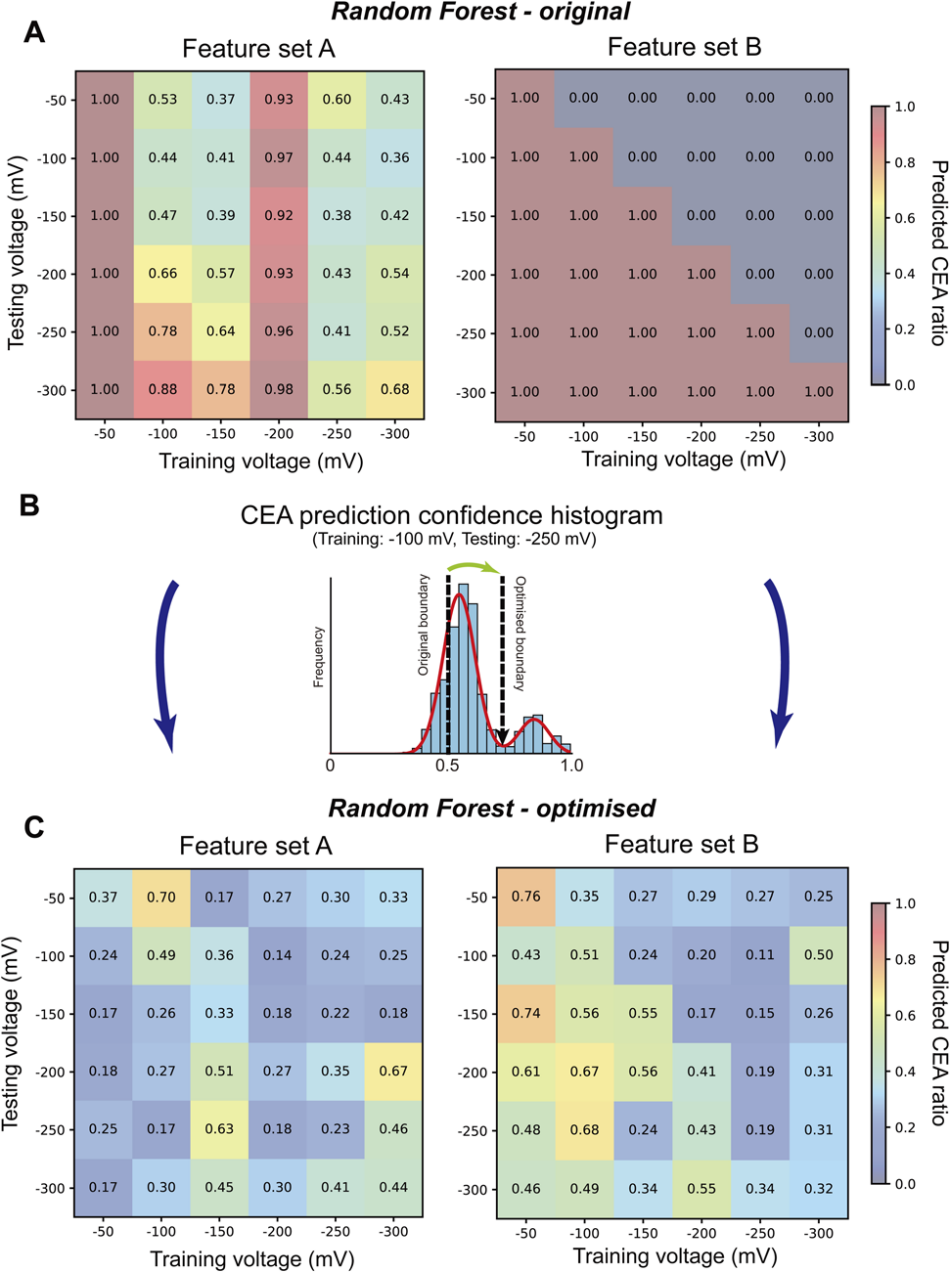
Optimizing CEA ratio predictions in voltage-matrix classification of mixed protein samples. (A) Heatmaps showing the predicted CEA ratio in 1 : 1 mixtures of CEA and CA15-3, obtained using RF classifiers trained on different voltages and tested on various voltages. Left and right panels correspond to feature sets A and B, respectively. (B) Representative histogram of prediction confidence scores (probability of CEA) from a model trained at −100 mV and tested at −250 mV. The initial threshold for CEA classification (0.5) results in a bimodal distribution. Two Gaussian distributions were fitted to these peaks, and the minimum point between them was selected as the optimized decision boundary. The updated ratio of predicted CEA signals was then calculated using the proportion of events exceeding this optimized threshold. (C) Voltage-matrix heatmaps of predicted CEA ratios after applying the optimized boundary adjustment shown in (B). The right panel (feature set B) shows improved signal rebalancing despite signs of overfitting in the original classification.

First, we applied the previously trained RF classifier to the mixed signals and evaluated the proportion of events classified as CEA. The resulting CEA classification ratios across the voltage matrix for both feature set A and feature set B are shown in Fig. 3A. In feature set A, except for the training voltages at −50 mV and −200 mV, higher predicted CEA ratios were observed in the lower-left portion of the matrix, where the training voltage was low and the testing voltage was high. Conversely, lower predicted CEA ratios were seen in the upper-right portion, where the training voltage was high and the testing voltage was low (Fig. 3A, left). This asymmetric classification behavior likely reflects voltage-dependent differences in capture frequency and translocation dynamics between CEA and CA15-3.^27^

In contrast, in feature set B, classification outcomes were polarized depending on the voltage combination, resulting in either 100% CEA or 100% CA15-3 predictions. These results are consistent with previous observations and are likely caused by overfitting to the baseline feature.

We next sought to interpret these results more precisely by examining the classification confidence across voltage conditions. For all combinations of training and testing voltages, we calculated histograms of predicted classification probabilities. In feature set A, most histograms exhibited two distinct peaks (Fig. S7), suggesting that the classification model successfully discriminated between CEA and CA15-3. Interestingly, however, in several specific voltage combinations, the two-peak distribution shifted toward either 0 or 1, while retaining the bimodal shape. Because classification was conducted using a 0.5 threshold, such shifts could lead to misclassification. Therefore, we fitted the two peaks with Gaussian distributions, estimated the optimal boundary value from the intersection, and recalculated the predicted CEA proportion based on the ratio of the peak areas (Fig. 3B). Similarly, for feature set B, where histograms were also skewed toward 0 or 1, we applied the same Gaussian fitting approach to extract corrected CEA prediction ratios (Fig. S8). We note that the observed deviations in predicted CEA ratios cannot be fully explained by capture frequency differences. In particular, the systematic deviation at −200 mV reflects voltage-dependent shifts in the shapes of event feature distributions rather than sampling imbalance alone. While capture frequency provides useful information about how often each analyte produces detectable events, it cannot be equated with the true prior probability used by the classifier. This is because acquisition filters (sampling rate, low-pass filtering) and detection thresholds can censor very fast or shallow events in an analyte- and voltage-dependent manner, so that the number of observed events does not directly represent the underlying molecular entry attempts (see Section 1.2 and 1.3 of the SI). To correct for these voltage-induced distribution shifts, we therefore apply histogram-based score distribution correction (Gaussian intersection; Fig. 3B, S7 and S8), treating capture frequency as informative but incomplete. To further illustrate the classification outcome at the single-event level, we provide scatter plots of the CEA–CA15-3 mixed samples after classification (Fig. S9). These plots show that the corrected probability-based classification yields consistent separation of event groups across different applied voltages. Interestingly, across all combinations of feature sets and voltage conditions, the corrected predicted ratios of CEA were generally confined to a range of approximately 0.2 to 0.6 (Fig. 3C). In this study, the mixtures were prepared at equal mass concentrations (2.0 μg mL^−1^ each), not equal molar concentrations. Given the difference in molecular weight between CEA and CA15-3, this means that the actual molar ratio favored CEA. From this perspective, corrected ratios below 0.5 are in fact consistent with the expected molecular composition. Moreover, in nanopore measurements, capture frequency is influenced not only by the number of molecules but also by physicochemical properties such as charge, size, and flexibility, which affect translocation under different voltage conditions. Therefore, deviations from the idealized 0.5 ratio should be interpreted as a combined outcome of the true molecular stoichiometry and intrinsic translocation properties, rather than as classifier inaccuracies. We also evaluated the classification performance using an SVM classifier. However, the model classified nearly all mixed-sample signals as CEA across all voltage conditions, likely due to model overfitting and sensitivity to subtle biases in the feature space. As a result, only the RF classifier results are shown in Fig. 3, reflecting its superior robustness across voltage variations. The present study demonstrates the feasibility of a voltage-matrix-based framework for the classification of protein signals acquired from SSNs. Through systematic comparisons across different voltage conditions, we uncovered characteristic trends in classification robustness and voltage sensitivity that depend on both the molecular identity and binding state of the analyte.

**Fig. 4:**
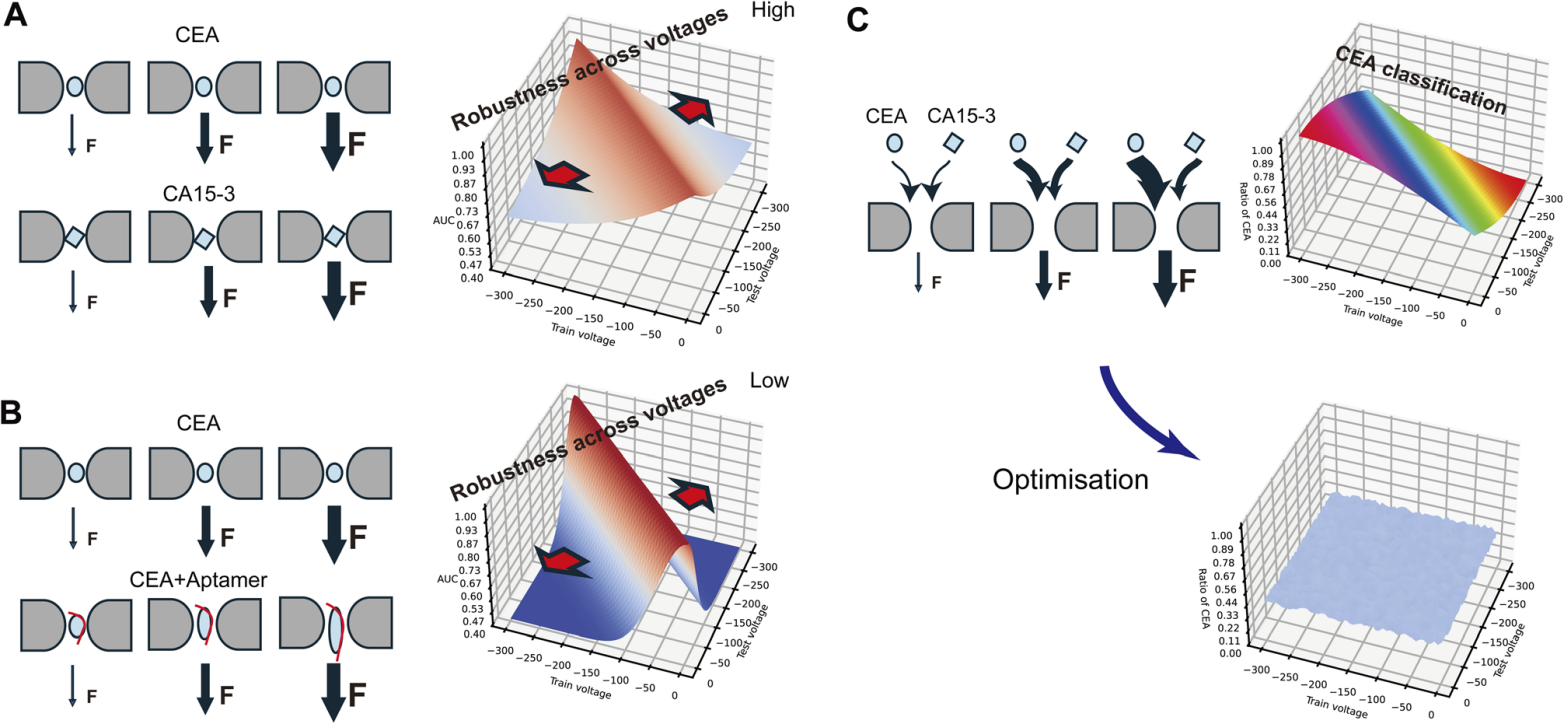
Conceptual model illustrating voltage-matrix-based discrimination and its optimization. (A) Classification between CEA and CA15-3 showed high AUC robustness across a range of training and testing voltages. This suggests that voltage-independent molecular properties—such as size and shape—primarily contributed to the discrimination. (B) In contrast, classification between CEA and the CEA–aptamer complex resulted in pronounced AUC drops when training and testing voltages differed, indicating that voltage-sensitive conformational changes were a major factor in classification performance. (C) Schematic overview of CEA classification in a 1 : 1 mixture of CEA and CA15-3. (Top right) Classifiers trained at different voltages estimated varying proportions of CEA under mismatched testing voltages, due to voltage-dependent capture biases. (Bottom right) After applying the prediction-boundary optimization strategy, the estimated CEA ratio became voltage-invariant, demonstrating improved robustness across voltage conditions. “F” indicates the external force applied to drive protein translocation through the nanopore in all schematic illustrations.

As visualized in Fig. 4A, CEA and CA15-3 exhibited consistent classification performance even when trained and tested at different voltages, suggesting that the classifier captures voltage-independent structural features—possibly size or conformation—that are preserved across conditions. In contrast, classification between CEA and the CEA–aptamer complex (Fig. 4B) revealed pronounced degradation in cross-voltage AUC, implying voltage-sensitive structural dynamics induced by aptamer binding. Previous reports support the notion that electric fields applied across nanopores can induce structural unfolding or rearrangement of protein complexes.^28^ These findings highlight how nanopore signal variability under different electrophoretic forces can reveal biomolecular interactions not readily captured under static conditions. Indeed, previous studies have successfully employed nanopores to probe protein structural changes, such as unfolding events, under different conditions.^29^

As illustrated in Fig. 4C, despite preparing a 1 : 1 mixture of CEA and CA15-3, the classifier's estimation of the proportion of CEA fluctuated significantly depending on the voltage condition. These results indicate that the observed capture frequency and signal characteristics are strongly influenced by voltage-sensitive physicochemical properties—such as charge, electrophoretic mobility, and structural flexibility—rather than being solely dictated by molar concentration. Notably, high CEA classification ratios were observed when the model was trained at low voltage and tested at high voltage, which can be interpreted as a result of the classifier being trained under conditions where CEA and CA15-3 had similar capture frequencies, and subsequently applied to conditions where CEA exhibited higher capture frequency.

Interestingly, the model trained at −50 mV classified nearly all mixed-sample signals as CEA regardless of the test voltage (Fig. 3A), and exhibited the lowest AUCs across voltage combinations (Fig. 1B). This behavior likely stems from the extremely low capture rate and poor translocation efficiency at −50 mV, leading to a narrowly biased subset of molecular signals used for training.

Previous studies have established that increasing the applied voltage enhances both the capture rate and translocation probability of molecules through nanopores, by amplifying the electrophoretic driving force that governs entry and passage.^8,27^ Although the absolute number of unsuccessful capture attempts may also increase at higher voltages due to stronger attraction to the pore entrance, the overall fraction of successful translocations tends to become dominant under elevated field strengths. Therefore, the observed voltage-dependent increase in event frequency in this study can be reasonably interpreted as a reflection of improved molecular throughput across the nanopore.

Under extremely weak electrophoretic force, only molecules with favourable size or charge characteristics can enter the nanopore, resulting in models trained under such conditions failing to generalize to the broader, more diverse molecular populations encountered at higher voltages.

While the cause of the high CEA classification ratio from the model trained at −200 mV remains unclear, it is plausible that the classifier captured voltage-specific signal characteristics unique to that condition. However, these effects were successfully compensated by recalculating the classification ratios using the prediction probability histograms, as described earlier.

This exemplifies how voltage acts not only as an analytical axis but also as a dynamic selector of molecular accessibility, underscoring the need for voltage-aware training strategies in nanopore sensing. This is consistent with reports showing the utility of nanopores for probing single-protein identity based on displacement sensing.^30^ These insights set the stage for exploring how additional orthogonal parameters can further enhance molecular discrimination in complex environments. This complexity echoes challenges found in conventional proteomic methods, such as two-dimensional gel electrophoresis, where molecules with similar molecular weights can be resolved using pH-dependent isoelectric focusing. In a similar manner, nanopore sensing can benefit from the incorporation of additional “axes” of separation to reveal subtle molecular differences. In this study, we demonstrated the utility of applied voltage as one such axis; however, future investigations involving more complex biological mixtures may require further parameters, such as pore diameter or buffer pH, to enhance resolution.

We next designed an experiment to evaluate whether the voltage-matrix framework can detect changes in molecular populations within complex biological samples. For this purpose, we compared native mouse serum before and after centrifugation, without spiking CEA or CA15-3. Classifiers trained *de novo* on serum data showed that classification accuracy (AUC) depended strongly on the applied voltage conditions; Random Forest provided the clearest separation when both training and testing at −100 mV and −150 mV (Fig. S10C), whereas other train–test combinations yielded only modest discrimination. Scatter plots of fractional blockage *versus* dwell time (Fig. S11) indicate that centrifugation alters the distribution of translocation event groups with distinct amplitude–duration characteristics—reducing long-dwell, shallow-amplitude events and shifting waveform statistics—consistent with the observed classification performance. These results suggest that, under specific voltage settings (−100/−150 mV), the classifier distinguishes the two serum preparations primarily by detecting redistribution of these event groups caused by centrifugation, rather than by identifying specific protein identities. The voltage-matrix framework thus demonstrates sensitivity to population-level changes in complex mixtures, although determining the molecular species responsible would require complementary techniques such as mass spectrometry or targeted immunoassays. In any case, further in-depth classification experiments and analyses are warranted, for which multidimensional profiling will be essential.

To enable such multidimensional profiling, the development of parallelized solid-state nanopore systems is essential. Simultaneous measurement across varied conditions will allow the construction of high-dimensional signal feature spaces and provide a path toward robust classification in clinical samples and real-world biosensing applications.

## Conclusions

In summary, we established a novel voltage-matrix framework for analysing and classifying nanopore signals generated by protein molecules and their complexes. Our findings revealed that applied voltage serves not only as a translocation driver but also as a dynamic modifier of signal features, which can be leveraged to distinguish subtle molecular differences.

The differential robustness across voltage conditions observed between unbound proteins and aptamer-bound complexes indicates the potential of this approach for probing biomolecular interactions. Furthermore, we demonstrated the feasibility of semi-quantitative molecular classification within mixed protein samples, a critical step toward real-world sample analysis.

Our results emphasize the importance of multi-parametric measurement strategies for decoding complex molecular ensembles. With continued advances in parallel nanopore technologies, the extension of this framework to broader molecular targets and multidimensional classification tasks is a promising direction for next-generation biosensing.

While we employed two classical machine learning models -RF and SVM- to evaluate voltage-matrix classification performance, our results revealed notable differences in their classification behavior. Specifically, the RF model tended to distribute feature importance more evenly, whereas SVM showed stronger dependence on a limited subset of features. These differences likely arise from their inherent learning mechanisms: RF aggregates decisions from multiple randomized trees, often capturing broader feature interactions, whereas SVM constructs a single optimal hyperplane and may rely heavily on features that contribute most to class separation in high-dimensional space. This model-dependent variance suggests that classification outcomes may be influenced not only by feature sets and voltage conditions but also by the choice of algorithm. Future studies incorporating additional classifiers or ensemble approaches may further enhance robustness across complex signal landscapes.^31^

## Author contributions

R. A., T. T., and S. U. conceived the study and designed the experiments. R. A., R. I., and S. U. discussed the experimental design and manuscript preparation. R. A. performed the experiments, established the analysis environment, and wrote the manuscript. A. L., K. A. B., T. Y., and D. K. performed data analysis. T. T., R. I., and S. U. supervised the experiments. S. U. coordinated the project and contributed to the acquisition of financial support. All authors discussed the results and contributed to the final manuscript.

## Conflicts of interest

There are no conflicts to declare.

## Supplementary Material

SC-OLF-D5SC05182G-s001

SC-OLF-D5SC05182G-s002

SC-OLF-D5SC05182G-s003

SC-OLF-D5SC05182G-s004

SC-OLF-D5SC05182G-s005

SC-OLF-D5SC05182G-s006

SC-OLF-D5SC05182G-s007

SC-OLF-D5SC05182G-s008

SC-OLF-D5SC05182G-s009

SC-OLF-D5SC05182G-s010

SC-OLF-D5SC05182G-s011

SC-OLF-D5SC05182G-s012

SC-OLF-D5SC05182G-s013

SC-OLF-D5SC05182G-s014

SC-OLF-D5SC05182G-s015

SC-OLF-D5SC05182G-s016

## Data Availability

All data are available in the main text or supplementary information (SI). Supplementary information is available. See DOI: https://doi.org/10.1039/d5sc05182g.
