# Supplementary material for: Voltage-matrix nanopore profiling for the discrimination of protein mixtures
Source: Chem Sci. 2025 Sep 23;16(40):18607–15. doi: 10.1039/d5sc05182g (PMC12499889; doi:10.1039/d5sc05182g)
Supplement: SC-016-D5SC05182G-s007 [file SC-016-D5SC05182G-s007.pdf]

Supplementary Information (SI) for Chemical Science.
This journal is © The Royal Society of Chemistry 2025

Electronic Supplementary Information

Voltage-Matrix Nanopore Profiling for the Discrimination of Protein Mixtures

Ryo Akita[a], Lysenko Artem[a], Keith A. Boroevich[b], Tatsuya Yokota[c], Daiki Kawai[a], Ryo Iizuka[a], Tatsuhiko Tsunoda[a,d] and Sotaro Uemura*[a]

[a] Ryo Akita, Lysenko Artem, Ryo Iizuka, Prof. Tatsuhiko Tsunoda, Prof. Sotaro Uemura
Department of Biological Sciences, Graduate School of Science, The University of Tokyo, 113-0033 (Japan)
E-mail: uemura@bs.s.u-tokyo.ac.jp
[b] Keith A. Boroevich
RIKEN Center for Integrative Medical Sciences, Yokohama 230-0045 Japan
[c] Tatsuya Yokota
Department of Computer Science, Nagoya Institute of Technology, 466-8555 Japan
[d] Prof. Tatsuhiko Tsunoda
Department of Computational Biology and Medical Sciences, Graduate School of Frontier Sciences, The University of Tokyo, 113-0033 Japan

Table of Contents

1. Experimental Procedures S3
    1.1 Sample Preparation S3
    1.2 Nanopore Measurements.. S3
    1.3 AFM Measurements.. S4
    1.4 Signal Events Extraction..S4
    1.5 Machine Learning Classification S5
2. Figures and Tables S7
3. References..S21

1. Experimental Methods

1.1 Sample Preparation

In this study, purified samples of carcinoembryonic antigen (CEA) and cancer antigen 15-3 (CA15-3) were used to evaluate the classification performance of solid-state nanopore detection in a controlled, non-biological matrix. Mouse serum was purchased from Bizcom Japan Inc. and subsequently used for centrifugation processing. CEA was purchased from Sigma-Aldrich, and CA15-3 from Sino Biological Japan. The DNA aptamer specific for CEA was synthesized according to a previously reported sequence [Ref 32]:

5′-
TCGCGCGAGTCGTCTGGGAGCTACGTTTAGCGAGTCCGACGCTCGGTGCCTC
TTCGGGAGGACGATGCGG-3′

All proteins and aptamers were diluted in 3.6 M LiCl / 10 mM HEPES-KOH buffer (pH 8.0) to ensure adequate signal-to-noise ratio in nanopore recordings. To assess voltage-dependent capture bias, a 1:1 mixture of CEA and CA15-3 (each at 2.0 μg/mL) was prepared. For aptamer–CEA complex formation, 1 μM aptamer was incubated with 10 nM CEA in the same buffer for 1 hour. The aptamer concentration was set at more than 10 times the reported dissociation constant (K_d = 37.8 ± 5.8 nM) to promote complex formation.

1.2 Nanopore Measurement

Controlled breakdown nanopore fabrication and pore-size estimation.

Nanopores were fabricated via controlled dielectric breakdown using Spark E2 (Northern Nanopore Instruments) according to the manufacturer's protocol. Silicon nitride membranes (12 ± 2 nm thick; Norcada Inc.) were mounted in electrolyte (1 M KCl / 10 mM HEPES-KOH, pH 8.0), and incremental DC voltage ramps were applied under current compliance until breakdown occurred, followed by immediate quenching to 0 V. Additional short voltage pulses were then applied in 3.6 M LiCl / 10 mM HEPES-KOH (pH 8.0) to enlarge the pore to a target open-pore conductance. Prior to recordings, brief conditioning (polarity-alternating pulses and low-bias flushing) was performed to stabilize the baseline. The pore diameter *d* was estimated to be ~12 nm from the open-pore conductance *G* (measured at the experiment temperature) using the cylinder-plus-access model,

$$R_{tot} = \frac{4L_{eff}}{\sigma\pi d^2} + \frac{1}{2\sigma d} \qquad G = \frac{1}{R_{tot}}$$

with solution conductivity σ measured under the same conditions. Here, R_{tot} represents the total ionic resistance (cylindrical pore resistance plus access resistance), and L_{eff} denotes the effective membrane thickness used in the cylindrical approximation. We confirmed low-bias I–V linearity and pre/post-run stability of G and I_0.

Nanopore measurement condition

Nanopore measurements were conducted using the SPARK-E2 device (Northern Nanopore Instruments). Voltage was controlled using an Axopatch 200B amplifier (Molecular Devices), with recordings performed at −50, −100, −150, −200, −250, and −300 mV. Each condition was measured for 5 minutes. Signals were sampled at 250 kHz and low-pass filtered at 10 kHz to suppress high-frequency noise.

1.3 AFM measurement

Atomic force microscopy (AFM) measurements were carried out using a Bruker scanning probe microscope (NanoScope V Dimension FastScan Bio) equipped with a SiN cantilever operated in PeakForce Tapping mode. Samples were prepared on ornithine-treated mica substrates and imaged in LiCl solution at room temperature. Scan sizes of 2 μm^2, 0.5 μm^2, and 0.2 μm^2 were collected at a scan rate below 1 Hz. For each sample, one representative region was imaged.

1.4 Signal Event Extraction

Current blockade events were automatically extracted using an algorithm combining AutoNanopore [Ref 33] and an exponentially weighted moving average (EWMA) baseline tracker, following established principles of adaptive baseline estimation [Ref 34, 35] AutoNanopore segments the raw ionic current trace into short intervals and applies extreme value theory (Gumbel distribution of maxima) to statistically model baseline fluctuations. This allows the detection threshold to be set in a data-driven and objective manner, avoiding the need for arbitrary multiples of standard deviation. By continuously updating the distribution of extreme noise amplitudes, AutoNanopore dynamically adapts to recording conditions and maintains high sensitivity to small events.

In parallel, the EWMA method adaptively follows the slowly drifting open-pore baseline and recalibrates the local noise level, ensuring robust detection under variable offsets and for long-duration signals. This combination provides several advantages: (i) adaptive thresholding based on noise statistics, (ii) robustness to baseline drift and slow fluctuations, and (iii) scalability to large datasets through automated processing without

manual intervention. These features enable consistent and reproducible event extraction across different voltages and experimental sessions, while minimizing user bias.

1.5 Machine Learning Classification

Machine learning was performed in Python 3.10.10 using scikit-learn (version 1.6.1).

Feature taxonomy and baseline dependence.

We categorized features a priori as event-intrinsic (baseline-agnostic) or baseline-dependent and used two sets accordingly. Set A exclusively uses normalized descriptors (e.g., fractional/normalized blockade, normalized area), whereas Set B additionally includes absolute-amplitude descriptors in pA (e.g., ΔI, absolute area) that carry baseline information (open-pore conductance, device/day noise). A complete list with definitions, units, classification, and set membership is provided in Supplementary Table S2.

Mixture-proportion estimation and prior adjustment

For mixture proportion estimation, we fitted bimodal histograms of prediction scores and used the Gaussian intersection as the decision threshold, which corrects threshold bias caused by voltage-dependent distribution shifts. Capture frequency was also considered as an approximate prior and applied in a sensitivity analysis using a log-prior boundary shift. However, systematic deviations such as those at −200 mV cannot be corrected by capture rate alone, since feature distributions themselves change with voltage. Therefore, we mainly report mixture estimates after score-distribution correction, while treating capture rate as informative but incomplete (see Results and the section 1.2-1.3 of the SI).

Model Training and Evaluation:

For each dataset, we first split the data randomly into training data (80%) and validation data (20%). To evaluate how well the models performed, we used the AUC (Area Under the Curve) calculated from the ROC curve. The ROC curve is a graph that shows how well the model separates different classes, and a higher AUC means better performance.

For the Random Forest (RF) model, we tuned the hyperparameters (the settings that control how the model works) using Grid Search, trying different combinations of the following values:

- n_estimators (number of trees): [10, 100, 200, 300]
- max_features (number of features to consider): ['sqrt']
- class_weight (class balancing method): ["balanced"]

- max_depth (maximum depth of trees): [5, 10, 50, None]

For the Support Vector Machine (SVM) model, we also used Grid Search with the following parameter ranges:

- C (penalty parameter for misclassification): [10, 100, 1000, 10000]
- gamma (controls the influence of each data point): [0.1, 0.01, 0.001, 0.0001]
- kernel (type of kernel function): ['rbf']

2. Figures and Tables

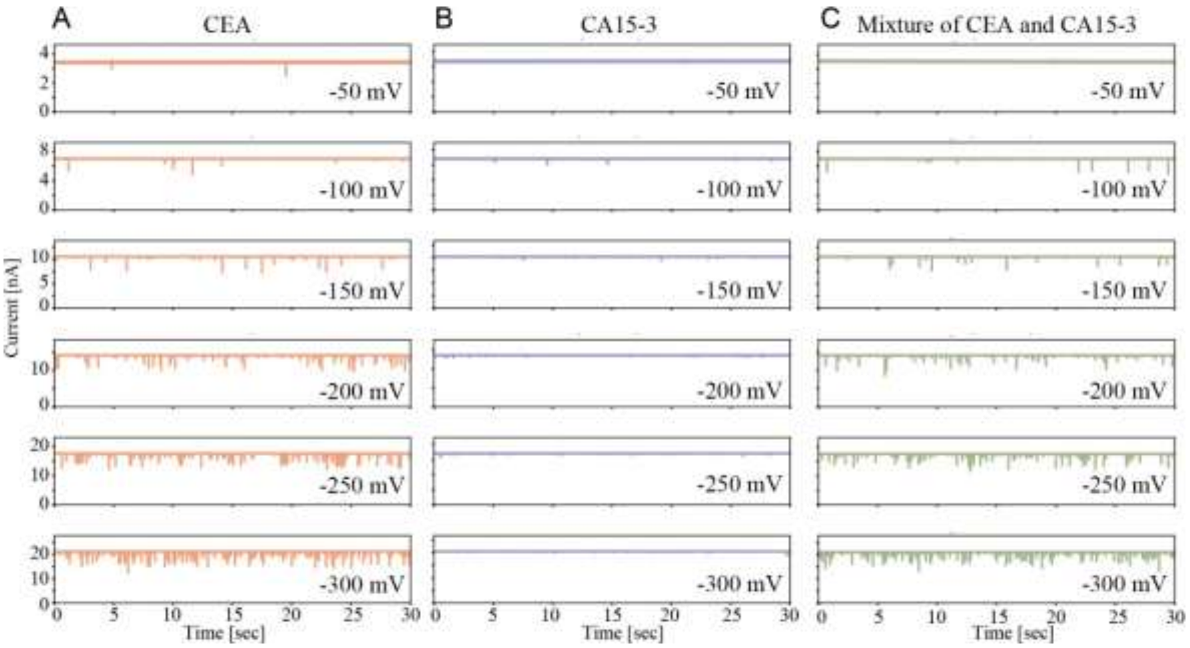

Fig S1. Representative nanopore ionic current traces recorded under six different applied voltages (−50 to −300 mV) for three sample types: (A) CEA only, (B) CA15-3 only, and (C) a 1:1 mixture of CEA and CA15-3.

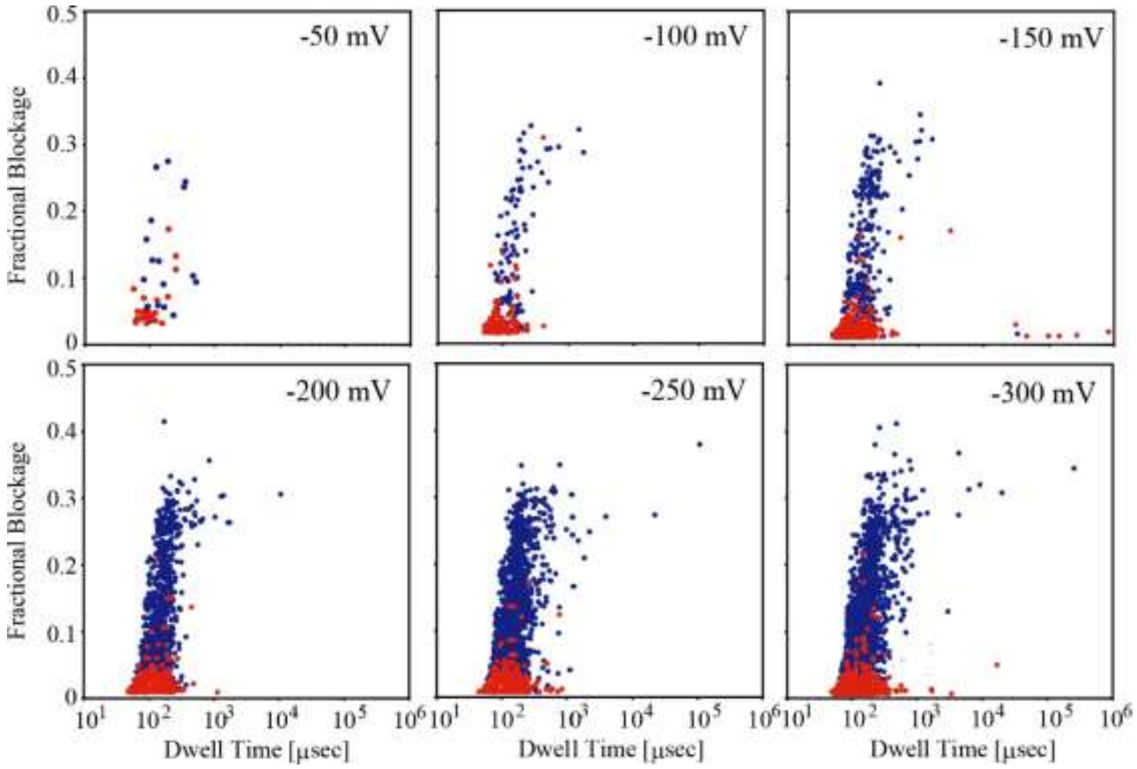

Fig S2. Two-dimensional scatter plots of extracted nanopore signals showing dwell time (horizontal axis, log scale) versus fractional current blockage (vertical axis) under six different applied voltages (−50 to −300 mV). Blue dots represent events from CEA-only samples, and red dots represent events from CA15-3-only samples.

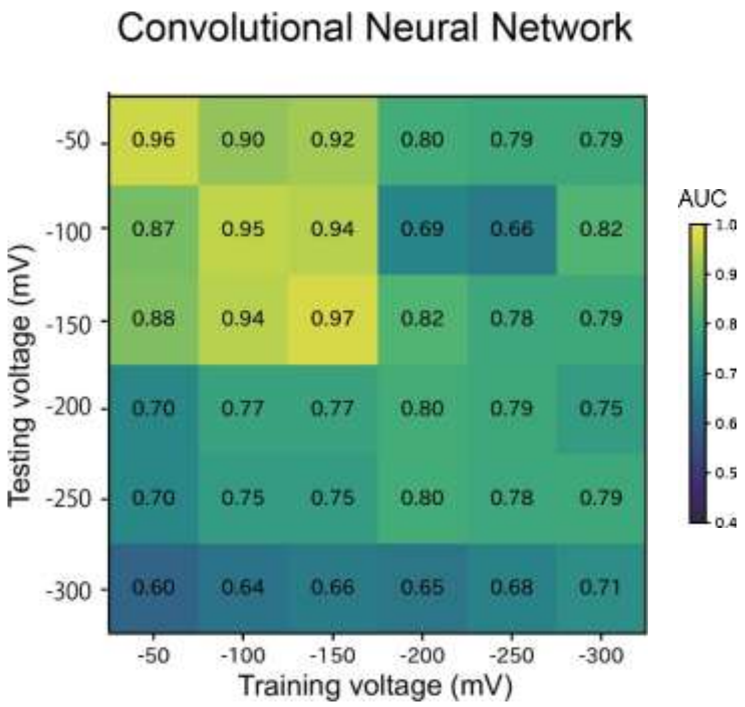

Fig S3. Voltage-matrix classification of CEA and CA15-3 using a CNN.
AUC values are shown for all combinations of training and testing voltages using CNN-based classification. CEA and CA15-3 signals were clearly separable across a wide voltage range, with consistently high AUC values (>0.9) in the region between −50 mV and −150 mV. This indicates that CNN models are capable of robustly capturing structural or kinetic features that differentiate the two proteins under various electric field conditions.

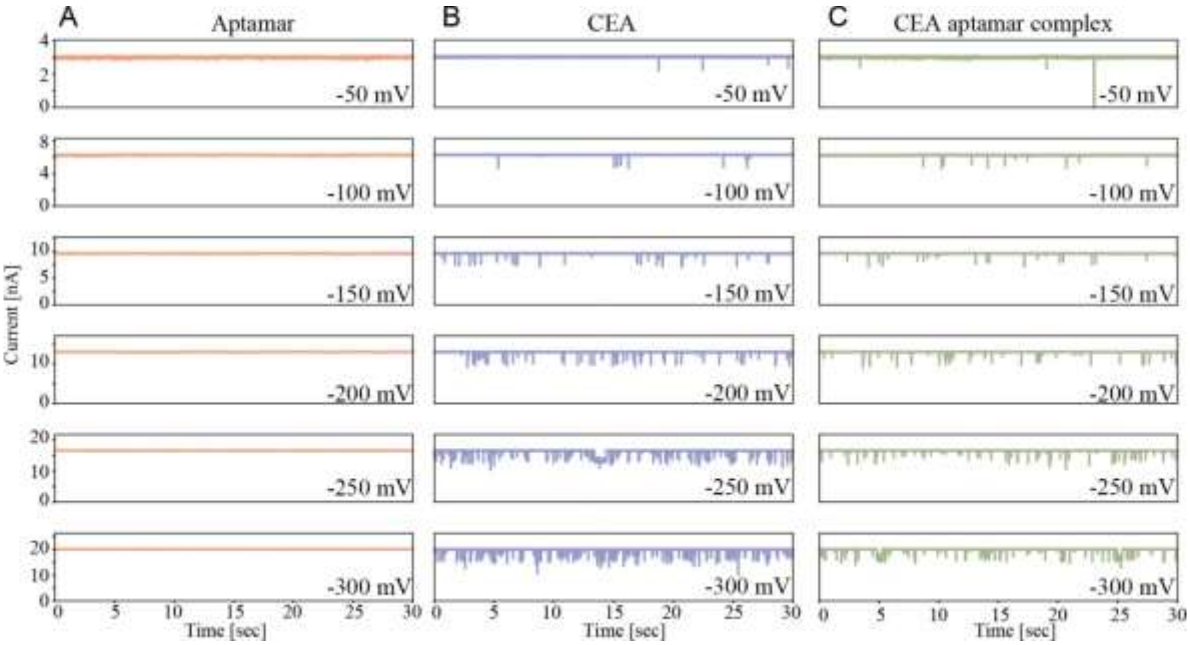

Fig S4. Representative nanopore current traces for three sample conditions under six applied voltages (−50 to −300 mV): (A) Aptamer only, (B) CEA only, and (C) CEA–aptamer complex.

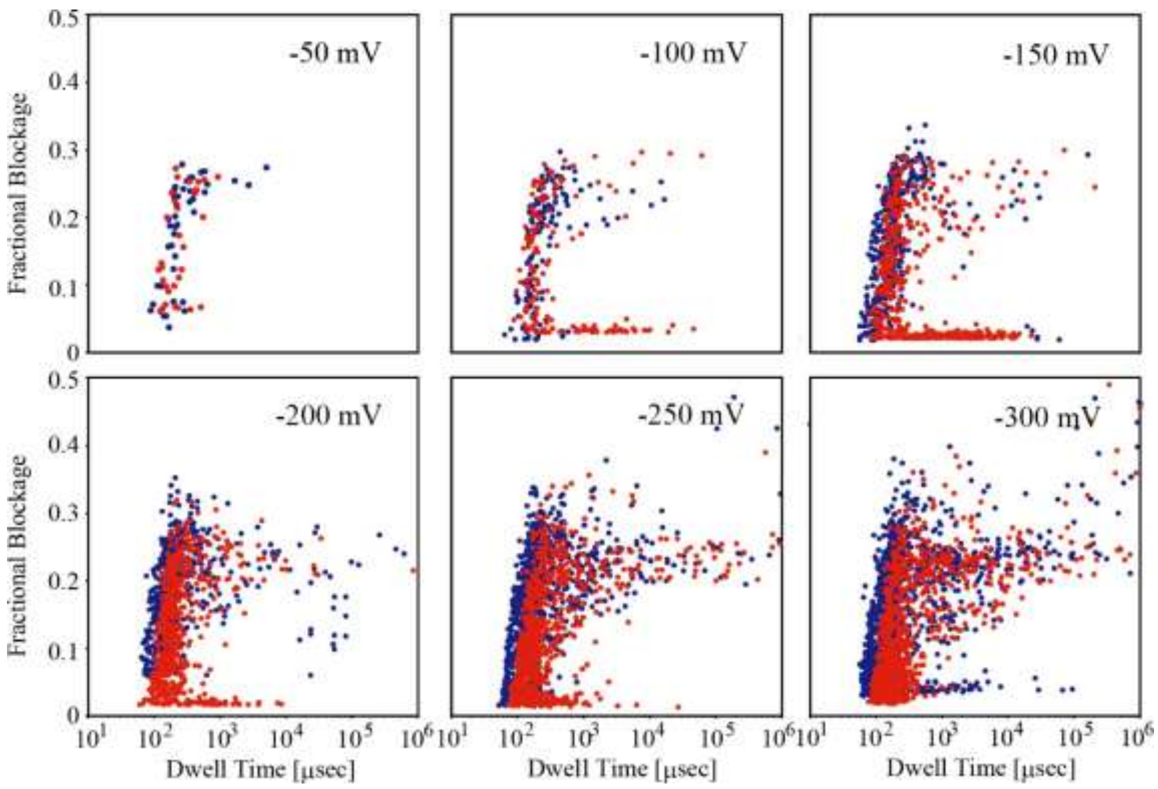

Fig S5. Two-dimensional scatter plots of nanopore translocation events, showing dwell time (horizontal axis, log scale) versus fractional current blockage (vertical axis) at six different applied voltages (−50 to −300 mV). Blue dots represent signals from CEA-only samples, while red dots represent signals from the CEA–aptamer complex.

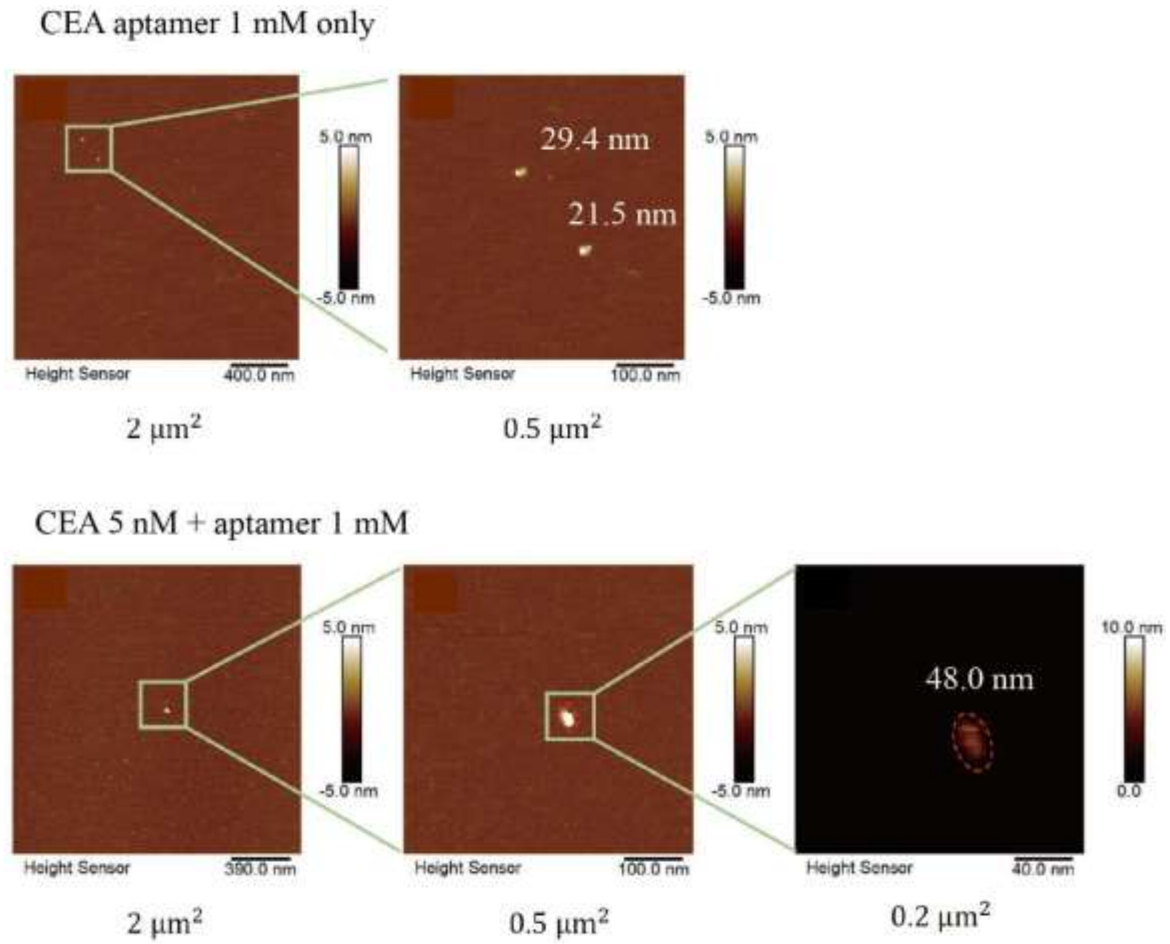

Fig S6. AFM images of aptamer alone and CEA–aptamer mixture. Representative AFM topographs obtained in PeakForce Tapping mode on ornithine-treated mica. Upper: DNA aptamer alone (1 µM), showing small particles with apparent diameters of ~20–30 nm. Lower: CEA (5 nM) mixed with aptamer (1 µM), showing larger structures with apparent diameters of ~40–50 nm. Absolute sizes appear larger than the expected molecular dimensions, which is commonly observed for nucleic acids in AFM due to substrate spreading and tip convolution.

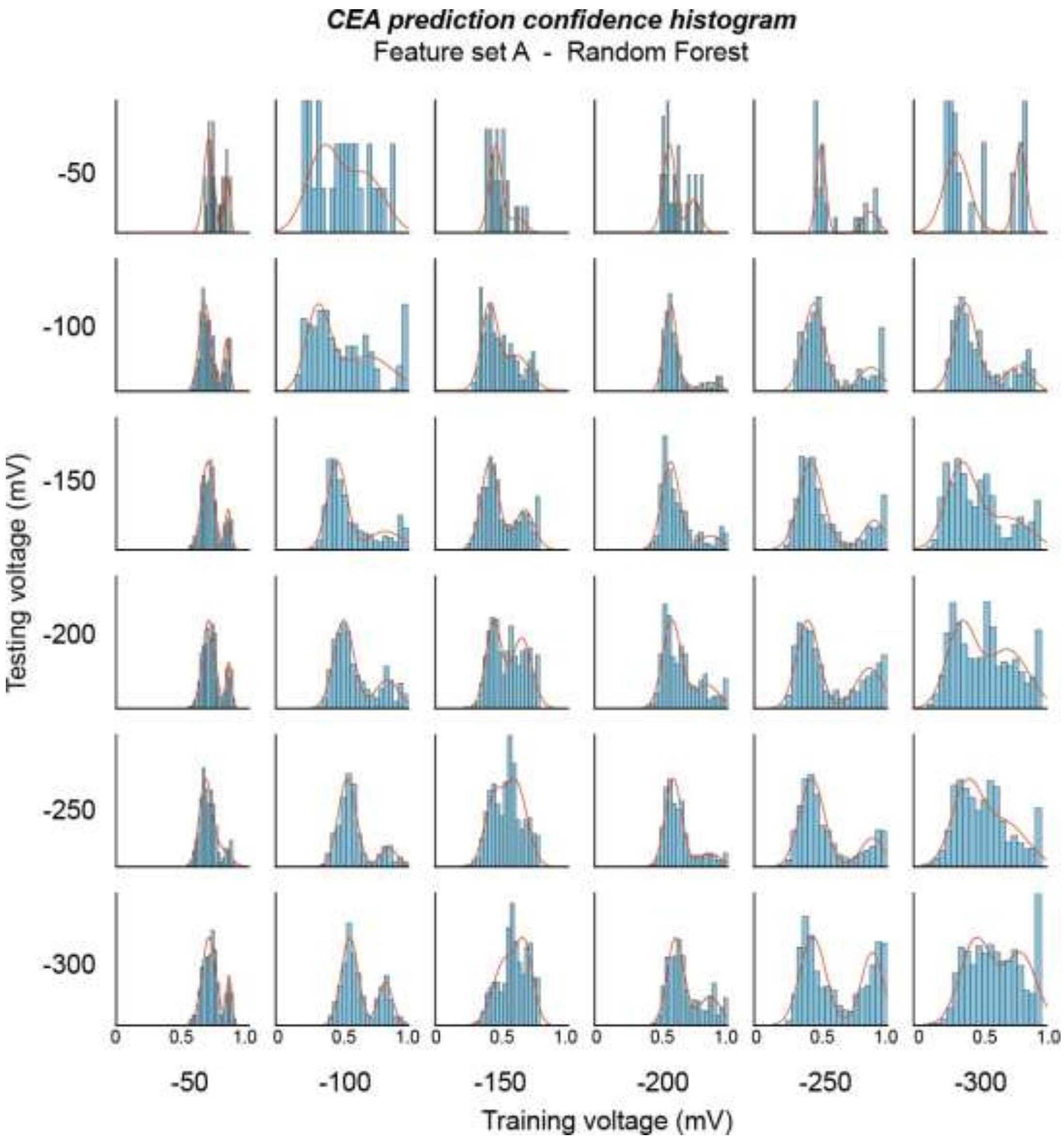

Fig S7. Prediction confidence histograms of CEA classification across voltage conditions using RF and Feature Set A.

Histograms show the distribution of prediction probabilities (CEA prediction confidence) for all combinations of training (x-axis) and testing (y-axis) voltages. Each subplot represents the classification outcome for a specific training–testing voltage pair, where the x-axis indicates prediction probability (0 to 1) and the y-axis indicates frequency. Overlaid red lines represent kernel density estimates to visualize bimodality. Many conditions exhibited bimodal distributions, suggesting successful separation of CEA and CA15-3 classes, while unimodal or skewed distributions highlight suboptimal separability under mismatched voltage conditions. These histograms served as the basis for the boundary optimization method described in Figure 3B.

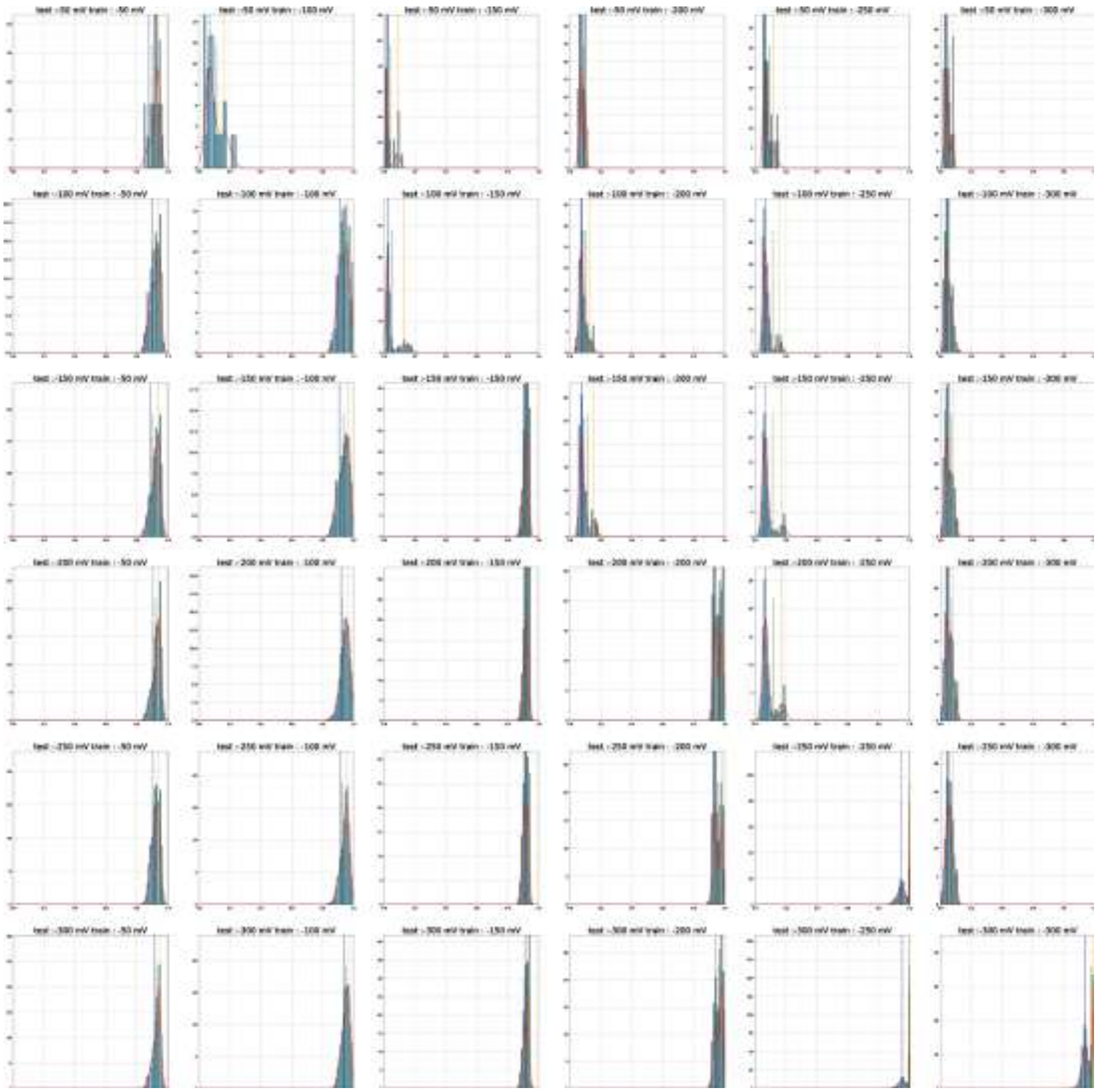

Fig S8. Prediction confidence histograms of CEA classification across voltage conditions using RF and Feature Set B.

Each subplot displays the distribution of predicted CEA probabilities for a specific pair of training and testing voltages. Histograms (blue bars) represent prediction confidence values (x-axis: 0 to 1), and red curves indicate kernel density estimates to reveal distribution patterns. Compared to Feature Set A (Figure S6), the distributions in Feature Set B are notably skewed or unimodal in most conditions, indicating lower robustness and limited class separability under voltage-shifted conditions. These results reflect a potential overfitting tendency of Feature Set B, further emphasized by the lack of clear bimodal patterns across most voltage combinations.

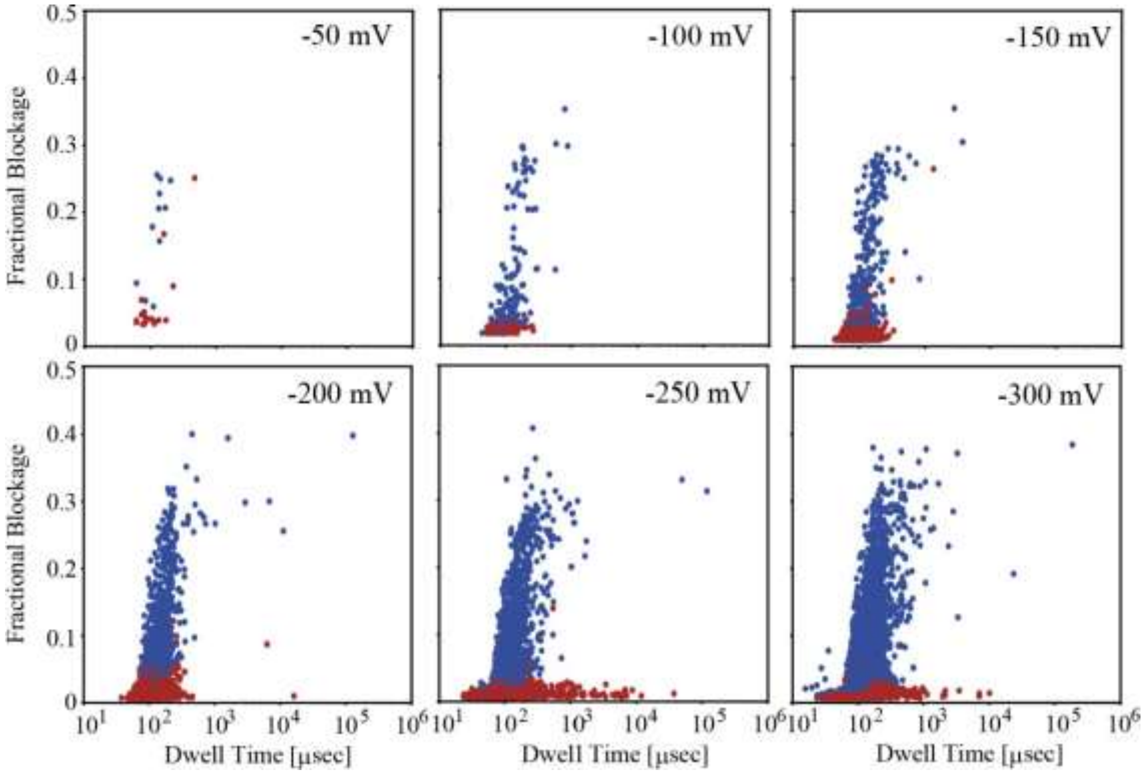

Fig S9. Scatter plots of CEA–CA15-3 mixed samples after classification.
Fractional blockage versus dwell time for mixed samples measured under different applied voltages. Data points were classified based on the probability histograms with correction, as described in the main text. Blue dots indicate events classified as CEA, and red dots indicate events classified as CA15-3.

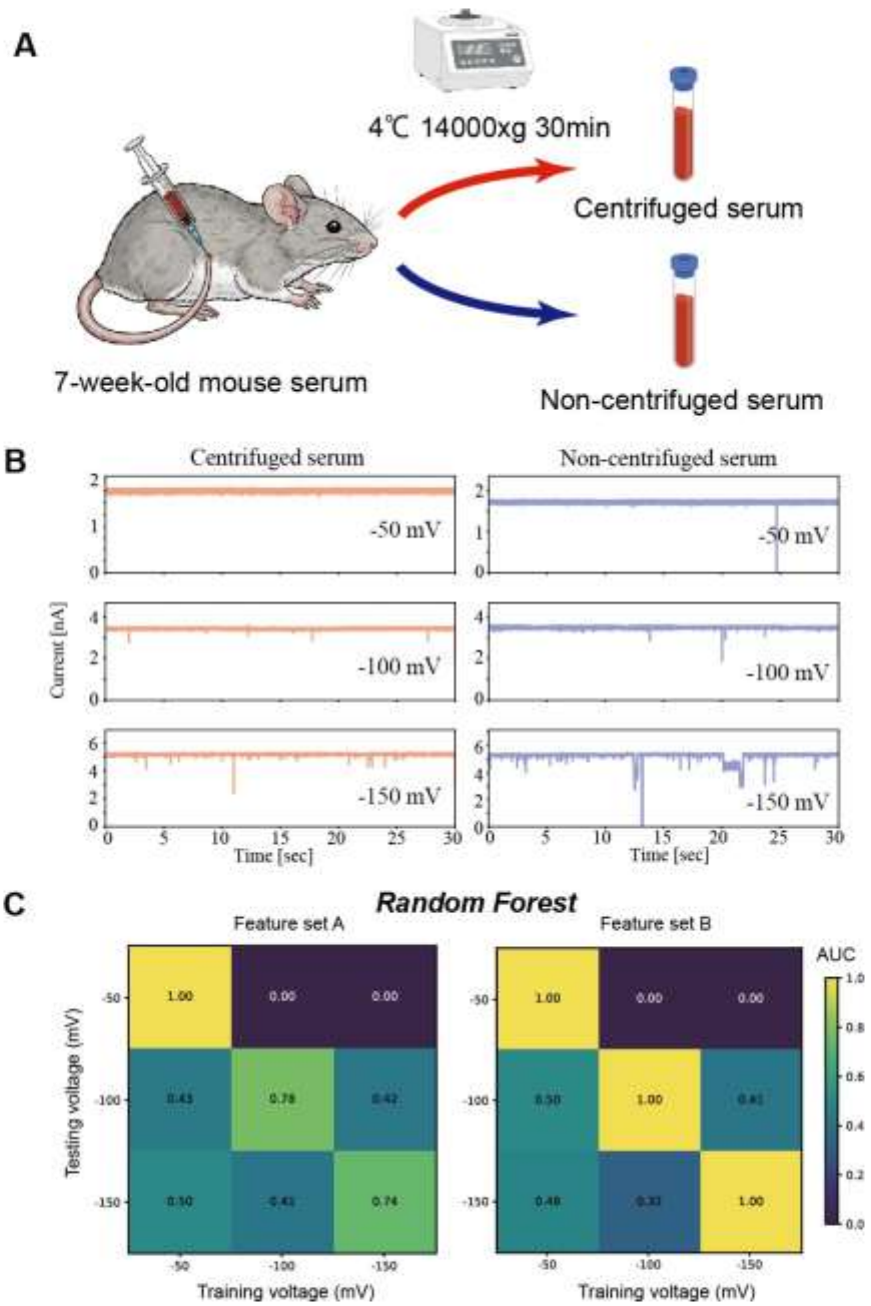

Fig S10. Voltage-matrix-based classification of mouse serum samples with and without centrifugation. (A) Schematic of sample preparation. Serum was collected from 7-week-old mice and either subjected to centrifugation at 4 °C, 14,000 × g for 30 minutes (centrifuged plasma) or used directly without processing (non-centrifuged plasma). The illustrations of the mouse, centrifuge, and tubes were generated using an AI-based image generation tool. (B) Representative ionic current traces obtained from nanopore measurements at −50, −100, and −150 mV. Event frequency and intensity were visibly higher in non-centrifuged serum. (C) AUC matrix maps representing classification performance between the two serum types using RF models. Feature set A (left) and feature set B (right) were used across different combinations of training and testing voltages.

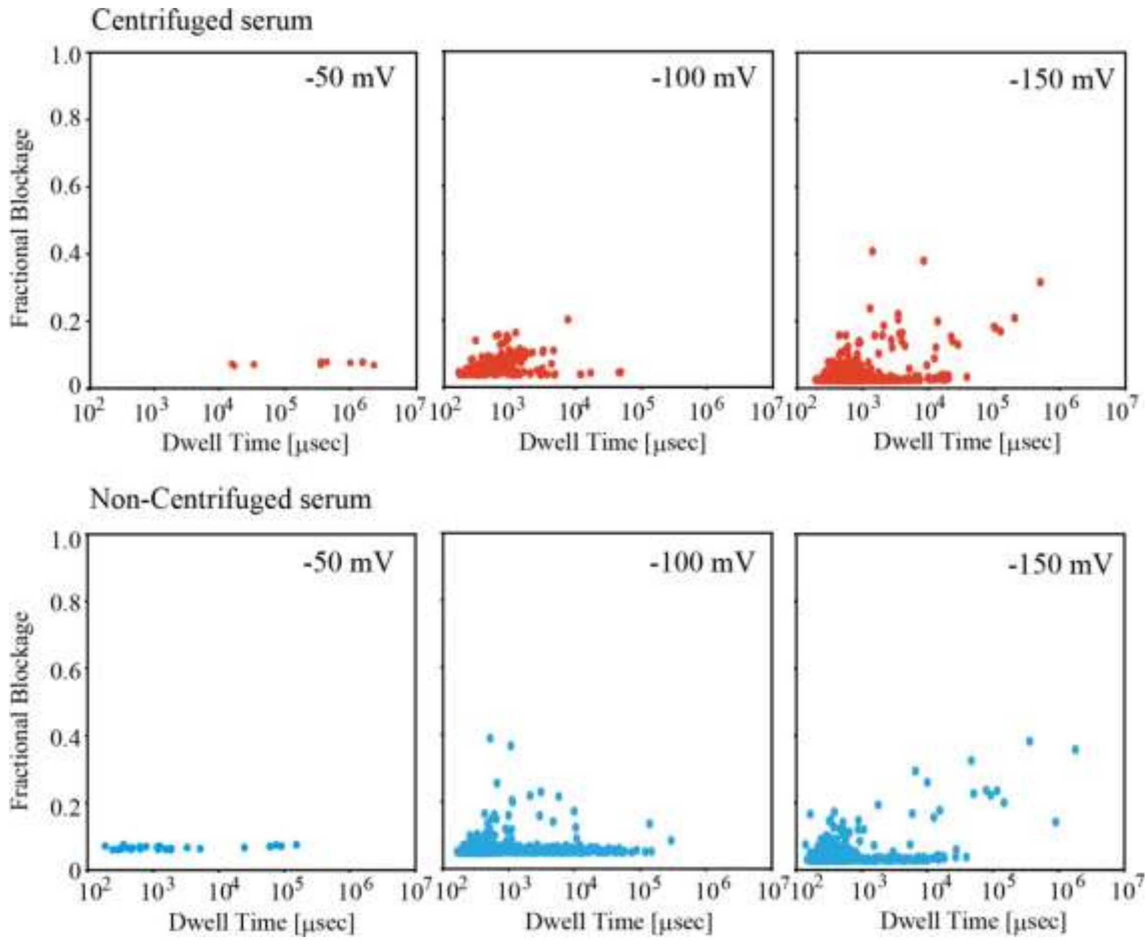

Fig S11. Scatter plots of serum samples with and without centrifugation.
Fractional blockage versus dwell time for mouse serum measured under different voltage conditions. Top row: centrifuged serum (−50, −100, −150 mV). Bottom row: non-centrifuged serum (−50, −100, −150 mV). Each dot represents a detected translocation event. Differences in event distributions highlight how centrifugation alters the detectable molecular populations in serum.

Table S1. Capture Frequencies of CEA and CA15-3 under Various Applied Voltages.

	-50mV	-100mV	-150mV	-200mV	-250mV	-300mV
CEA	4.1	26.3	89.6	203.1	347.1	439.5
CA15-3	4.4	28.2	96.0	172.5	231.9	244.7
Sum	8.5	54.6	185.6	375.6	578.9	684.1
1:1 Mixture	6.2	61.4	183.9	396.3	979.1	961.1

Capture frequencies (events/min) were measured for individual CEA and CA15-3 samples, along with their 1:1 mixture, at six different voltage conditions.

Table S2. Classification of features used in this study (event-intrinsic vs baseline-dependent)

Feature name	Symbol / Definition	Used in set	Classified as	Rationale
Normalized max blockage	$(\Delta I_{max})/I_0$	A	Event-intrinsic	Normalized by baseline; reflects instantaneous occlusion.
Normalized average blockage	$(\Delta I)/I_0$	A	Event-intrinsic	Normalized amplitude; independent of absolute baseline.
Duration (τ)	Event length (ms)	A & B	Event-intrinsic	Reflects passage kinetics; not baseline-dependent.
Peak position	Relative index of minimum within event window	A & B	Event-intrinsic	Geometry of waveform; independent of baseline.
Area	$\int \Delta I(t)dt$; normalized in A, absolute in B	A & B	Mixed	Normalized: intrinsic waveform; absolute: scales with I_0.
Left slope	Ingress slope dI/dt	A & B	Event-intrinsic	Entry dynamics; waveform-intrinsic.
Right slope	Egress slope dI/dt	A & B	Event-intrinsic	Exit dynamics; waveform-intrinsic.
Standard deviation (within-event)	SD of event current (normalized or absolute)	A & B	Event-intrinsic	Shape/noise metric; independent after normalization.
Kurtosis	Excess kurtosis of event waveform	A & B	Event-intrinsic	Higher-order waveform shape descriptor.
Skewness	Skewness of event waveform	A & B	Event-intrinsic	Asymmetry of blockade waveform.
Full width at half maximum (FWHM)	Duration above 50% of max blockade	A & B	Event-intrinsic	Width of main blockade region; independent of baseline.
Max blockage (absolute)	$\Delta I_{max}=I_0$ - min I(t) (pA)	B	Baseline -dependent	Directly scales with I_0; condition-specific.
Average blockage (absolute)	ΔI (pA)	B	Baseline -dependent	As above; tied to pore conductance.
Ratio of block feature	$\frac{\text{Blockage}}{\text{Normalized blockage}} \approx I_0$	B	Baseline -dependent	Effectively encodes open-pore current; a device signature.

Notes.
I_0: open-pore current immediately before each event (median over a short pre-event window).

Table S3. Capture Frequencies of Aptamer, CEA and CEA-Aptamer complex under Various Applied Voltages.

	-50mV	-100mV	-150mV	-200mV	-250mV	-300mV
Aptamer	0.0	0.0	0.0	0.0	0.0	0.0
CEA	8.5	30	75	120	200	290
CEA-Aptamer complex	6.8	30	100	110	160	170

Capture frequencies (events/min) were measured for individual Aptamer and CEA samples, along with CEA-Aptamer complex, at six different voltage conditions.

3. References

32. de Melo, M. I. A.; Correa, C. R.; Cunha, P. d. S.; de Góes, A. M.; Gomes, D. A.; Andrade, A. S. R. DNA aptamers selection for carcinoembryonic antigen (CEA). *Bioorg. Med. Chem. Lett.* **2020**, *30*, 127278, DOI: 10.1016/j.bmcl.2020.127278.
33. Sun, Z.; Kamakoti, R.; Wilson, J.; Lee, J. S. AutoNanopore: A Fast and Automated Event Extraction Method for Nanopore Sensing Based on Extreme Value Theory. *ACS Omega* **2022**, *7*, 24591–24600. DOI: 10.1021/acsomega.2c02927
34. Roberts, S. W. Control charts using exponentially weighted moving averages, *Technometrics*, **1959**, 1, 239–250. DOI: 10.1080/00401706.1959.10489860.
35. Lucas, J. M.; Saccucci, M. S. Exponentially weighted moving average control schemes: properties and enhancements, *Technometrics*, **1990**, 32, 1–12. DOI: 10.1080/00401706.1990.10484960.
